# Ouabain Modulates the Functional Interaction Between Na,K-ATPase and NMDA Receptor

**DOI:** 10.1007/s12035-020-01984-5

**Published:** 2020-07-10

**Authors:** Evgeny E. Akkuratov, Linda Westin, Erika Vazquez-Juarez, Minttu de Marothy, Aleksandra K. Melnikova, Hans Blom, Maria Lindskog, Hjalmar Brismar, Anita Aperia

**Affiliations:** 1 Science for Life Laboratory, Department of Applied Physics, Kungliga Tekniska Högskolan, Stockholm, Sweden; 2 Science for Life Laboratory, Department of Women’s and Children’s health, Karolinska Institutet, Stockholm, Sweden; 3 Department of Neurobiology, Care Sciences and Society, Karolinska Institutet, Stockholm, Sweden; 4 Faculty of Bioengineering and Bioinformatics, Lomonosov Moscow State University, Moscow, Russia 119234

**Keywords:** Na,K-ATPase, NMDAR, Ouabain, Calcium, LTP, Super-resolution microscopy, Protein-protein interaction

## Abstract

The N-methyl-D-aspartate (NMDA) receptor plays an essential role in glutamatergic transmission and synaptic plasticity and researchers are seeking for different modulators of NMDA receptor function. One possible mechanism for its regulation could be through adjacent membrane proteins. NMDA receptors coprecipitate with Na,K-ATPase, indicating a potential interaction of these two proteins. Ouabain, a mammalian cardiotonic steroid that specifically binds to Na,K-ATPase and affects its conformation, can protect from some toxic effects of NMDA receptor activation. Here we have examined whether NMDA receptor activity and downstream effects can be modulated by physiological ouabain concentrations. The spatial colocalization between NMDA receptors and the Na,K-ATPase catalytic subunits on dendrites of cultured rat hippocampal neurons was analyzed with super-resolution dSTORM microscopy. The functional interaction was analyzed with calcium imaging of single hippocampal neurons exposed to 10 μM NMDA in presence and absence of ouabain and by determination of the ouabain effect on NMDA receptor–dependent long-term potentiation. We show that NMDA receptors and the Na,K-ATPase catalytic subunits alpha1 and alpha3 exist in same protein complex and that ouabain in nanomolar concentration consistently reduces the calcium response to NMDA. Downregulation of the NMDA response is not associated with internalization of the receptor or with alterations in its state of Src phosphorylation. Ouabain in nanomolar concentration elicits a long-term potentiation response. Our findings suggest that ouabain binding to a fraction of Na,K-ATPase molecules that cluster with the NMDA receptors will, via a conformational effect on the NMDA receptors, cause moderate but consistent reduction of NMDA receptor response at synaptic activation.

**Electronic supplementary material:**

The online version of this article (10.1007/s12035-020-01984-5) contains supplementary material, which is available to authorized users.

## Introduction

The N-methyl-D-aspartate (NMDA) receptor plays an essential role in formulation of synaptic plasticity and memory [1]. In contrast to the AMPA-type of glutamate receptors at the mature synapse, NMDA receptors are permeable to calcium, and the calcium signal mediated by NMDA receptor activation is a major determinant of synaptic strength and propensity to potentiate. The NMDA receptor is involved in the modulation of many functions, including memory and mood. Reduction of NMDA receptor activity has recently been suggested as a target for antidepressant treatment [2]. NMDA receptor activity can also have deleterious effects, and calcium-dependent excitotoxicity is a common cause of cell death in stroke [3, 4].

It was recently shown that the NMDA receptor may interact with the salt pump, Na^+^, K^+^-ATPase (NKA) [5, 6]. NKA transports three sodium ions out of the cell and two potassium ions into the cell at the expense of 1 molecule ATP and is responsible for the electrochemical gradient across the cell membrane and the restoration of the intracellular sodium concentration to basal level after neuronal activation [7]. NKA-mediated ion transport accounts for approximately 50% of total brain energy consumption [8].

NKA has a highly specific binding site for cardiotonic steroids (CTS). Several CTS, including ouabain, have been identified in mammals [9, 10] and shown to be produced in the adrenals and the hypothalamus [11]. NKA exhibits a biphasic response to ouabain. Micromolar concentrations of ouabain saturates NKA in rat and inhibits its ion pumping function with immediate effects on cellular ion balance. Nanomolar ouabain concentrations has no immediate effect on cell ion homeostasis but can initiate a signal function. NKA signaling has been extensively studied in epithelial cells and cardiomyocytes, where treatment with ouabain in subsaturating concentrations can activate Src phosphorylation [12], calcium oscillations [13], and a large scale signaling network [14]. In the brain, ouabain treatment has been reported to improve the behavioral outcome in a model of traumatic brain injury [15], and intrathecal administration of ouabain has been shown to protect from apoptosis in rats with ischemic stroke [16] and excitotoxicity [17].

In this study, we have explored the conditions for a functional crosstalk between the NMDA receptor and ouabain exposed NKA in hippocampal neurons. To test the effect of ouabain on NMDA receptor calcium signaling, we used a microfluidic device where NMDA can be applied either alone or together with a subsaturating concentration of ouabain in a highly restricted region of a single cell. This approach allowed us to describe a local, immediate, and rapidly reversible ouabain regulation of the NMDA-mediated calcium response. Colocalization of NKA and NMDA receptor proteins in intact neurons was demonstrated with two complementary methods, the proximity ligation assay and super-resolution STORM microscopy. Since our results indicated a close colocalization between NKA and the NMDA receptor, we tested the functional importance of an interaction by determining the effect of subsaturating ouabain concentrations on long-term potentiation.

## Results

### Ouabain Attenuates NMDA-Mediated Calcium Influx in Hippocampal Neurons

**Fig. 1:**
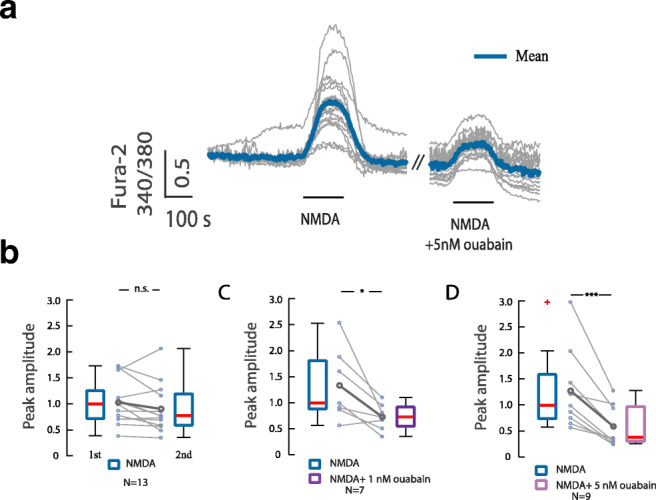
Calcium imaging of NMDA evoked responses in hippocampal neurons in absence and presence of ouabain. **a** Traces of calcium responses in cells exposed to NMDA (4 μM) without and with ouabain (5 nM) for 2 min. **b** Calcium response upon subsequent NMDA stimulation of the same cell. **c** Calcium response of NMDA treatment alone and together with ouabain (1 nM). **d** Calcium response of NMDA treatment alone and together with ouabain (5 nM). Boxplots show the peak amplitude normalized to median of peak 1 amplitudes. n.s.—*p* > 0.05, *p < 0.05, ***p < 0.001, Wilcoxon signed-rank test

We first tested whether the NMDA-mediated calcium response can be modified by subsaturating concentrations of ouabain in primary hippocampal neurons using a perfusion system (Fig. 1). When NMDA (4 μM) was added to the cells for 2 min, a rapid and reversible increase in intracellular calcium was observed. Application of NMDA together with ouabain (1 or 5 nM) significantly reduced the calcium response. There was no change in calcium responses between the first and second application when NMDA was added alone.

**Fig. 2:**
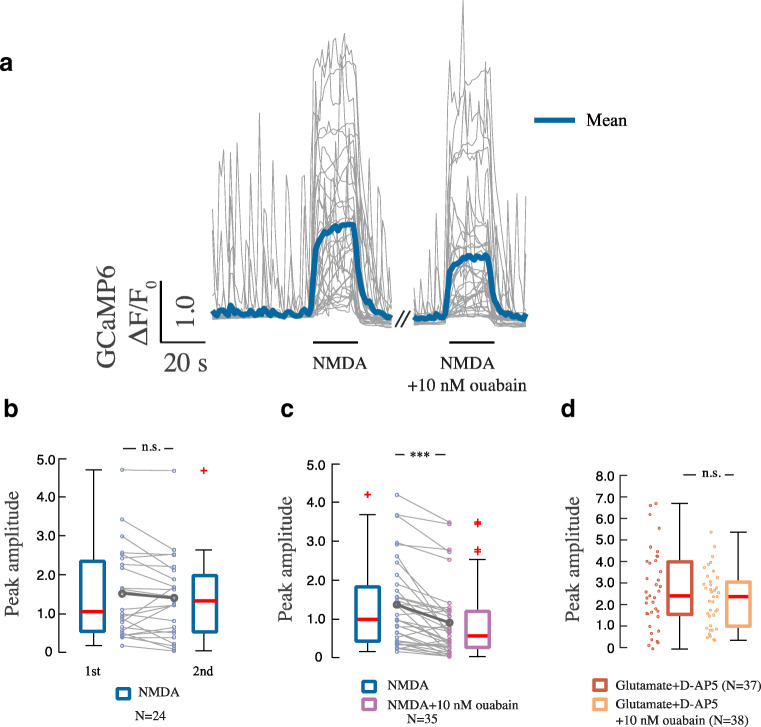
Rapid effect of nanomolar concentrations of ouabain on NMDAR-dependent calcium influx by local stimulation. **a** Traces of calcium responses in cells exposed to NMDA (10 μM) without and with ouabain (10 nM) for 20 s. Same concentrations apply in **b** and **c**. **b** Calcium response in cells exposed to subsequent 20-s pulses of NMDA. Boxplots show mean ΔF/F0 for the first and second NMDA application. **c** Boxplot presentation over ΔF/F0 upon stimulation with NMDA alone and respective NMDA + ouabain. n.s.—*p* > 0.05, ***p < 0.001, Wilcoxon signed-rank test. **d** Boxplot shows calcium responses during 20-s treatments with glutamate (10 μM) + D-AP5 (50 μM) and glutamate + D-AP5 + ouabain (10 nM). n.s.—*p* > 0.05, Wilcoxon rank sum test

Next we tested the ouabain effect on the NMDA-mediated calcium response in single neurons to exclude an indirect network effect using a microfluidic device where solutions loaded in the pipette could, through convective recirculation, be applied to a single neuron (Supp. Fig. 1, Supp. Video 1). For the calcium recordings, we used the calcium-sensitive sensor GCaMP6f [18], which was applied with low transfection efficacy to get good resolution of single dendrites. Addition of NMDA (10 μM) for 20 s to a single GCaMP6f-expressing neuron produced a rapid and reproducible increase in cytosolic calcium (Fig. 2a–c). When NMDA was applied together with ouabain (10 nM), the calcium response was significantly reduced. Repeated treatment with NMDA (10 μM) alone did not alter the magnitude of the calcium response.

To test whether the ouabain effect involved other ionotropic glutamate receptors, we repeated the experiments using glutamate (10 μM) together with D-AP5 (50 μM), a competitive NMDA receptor antagonist [19]. Under these conditions, the calcium response was not attenuated by ouabain (Fig. 2d). Ouabain (10 nM or 1 μM) alone did not cause changes in resting calcium level (Supp. Fig. 2). In addition, ouabain (given in concentrations from 1 nM to 1 μM) did not affect the membrane potential (Supp. Fig. 3). Thus, we show that ouabain in concentrations 1–10 nM specifically reduces the NMDA-mediated calcium signal in a cell-autonomous manner.

### Ouabain Downregulation of NMDA Response Is Not Dependent on NMDA Receptor Internalization or Src Phosphorylation

**Fig. 3:**
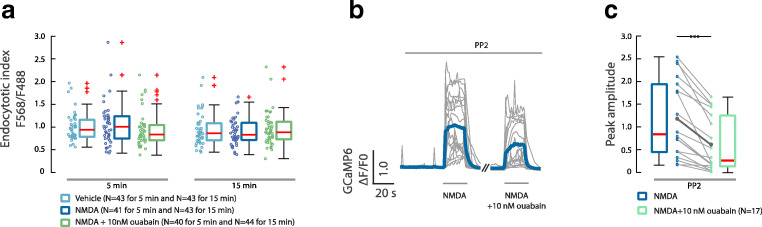
The ouabain effect on NMDAR-mediated calcium response is not due to internalization of NMDAR or Src-activation. **a** Comparison between cytosolic and membrane expression of NMDAR in the presence of NMDA (10 μM) alone and with ouabain (10 nM). The endocytotic index was measured in cells treated for 5 min and 15 min. One-way ANOVA and Bonferroni’s post hoc test were used. **b** Traces of calcium responses in cells treated for 20 s with NMDA (10 mΜ) alone and with ouabain (10 nM), in the presence of PP2 (1 μM). **c** Boxplot presentation over ΔF/F0 upon stimulation with NMDA and respective NMDA + ouabain. ***p < 0.001, Wilcoxon signed-rank test

NMDA receptors are generally considered to be stable components of the postsynaptic density, but in primary neuronal cultures early in development, NMDA receptor have been reported to undergo rapid internalization [20, 21]. Since ouabain has also been reported to stimulate endocytosis of AMPA receptors [22], we tested whether exposure to nanomolar concentrations of ouabain might result in internalization of the GluN2B subunit of NMDA receptor using method described by Kopec et al. (Supp. Fig. 4) [23]. Application of NMDA alone or with co-application with ouabain did not change the endocytotic index compared with control (Fig. 3a).

The activity of the NMDA receptor is regulated by posttranslational modification including Src-mediated phosphorylation [24]. The Src family tyrosine kinases (SFK) potentiate NMDA receptor activity and increase the gating of the NMDA receptor channel [25]. Since the ouabain-bound NKA has been reported to activate Src kinase [12], we tested whether the SFK inhibition with PP2 (10 μM) would abolish ouabain-dependent downregulation of the NMDA receptor induced calcium response. We found that inhibition of SFK activity after pretreatment with PP2 (1 μM) did not abolish the effect of ouabain (10 nM) on NMDA receptor–dependent calcium influx (Fig. 3b, c).

**Fig. 4:**
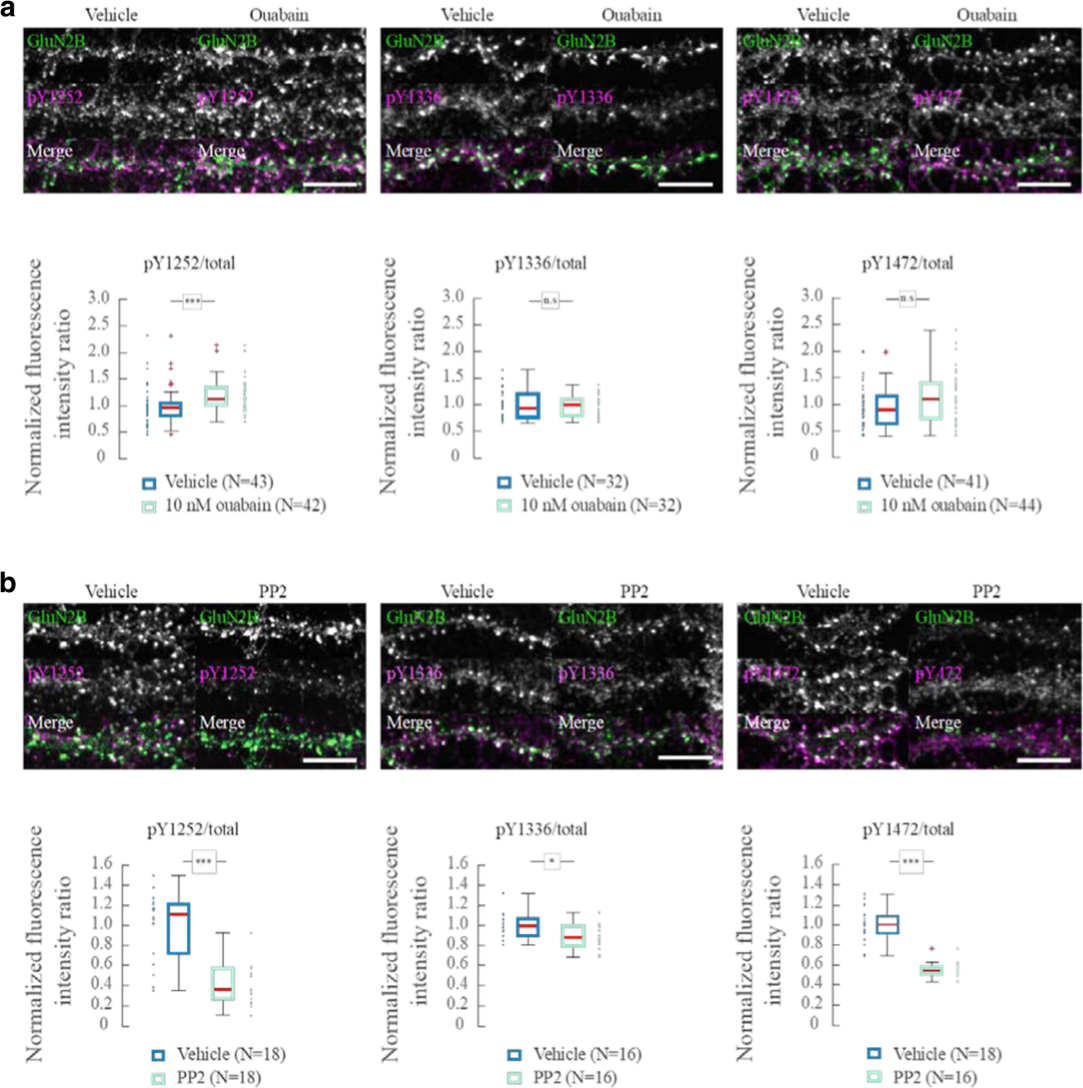
Tyrosine phosphorylation of GluN2B. **a** Antibody labeling of the three SFK phosphorylation sites of GluN2B: pY1252, pY1336, and pY1472, and total GluN2B in dendrites following 5 min application of vehicle or 10 nM ouabain. Boxplots of the ratio of the fluorescent intensity of pYGluN2B/GluN2Btotal normalized to the mean under control conditions with vehicle. **b** As in **a** but exposed to DMSO (vehicle) or 10 μM PP2 for 5 min. Boxplots of the ratio of the fluorescent intensity of pYGluN2B/GluN2B total normalized to the mean under control conditions with DMSO. n.s.—*p* > 0.05, *p < 0.05, ***p < 0.001, Wilcoxon rank sum test. Scale bar = 10 μm

We also studied the effect of ouabain treatment on the three sites of GluN2B that are phosphorylated by SFK (pY1252, pY1336, and pY1472) using phosphorylation-specific antibodies. We found a small increase in the phosphorylation of Y1252 in ouabain-treated neurons. There was no significant difference in the level of phosphorylation of Y1336 and Y1472 between ouabain-treated and control neurons. When the cultured neurons were exposed to PP2 (10 μM), phosphorylation of pY1252, pY1336, and pY1472 decreased, demonstrating that NMDAR are constitutively phosphorylated in the culture (Fig. 4).

### NKA and NMDA Receptors Exist in Close Proximity in Hippocampal Neurons

**Fig. 5:**
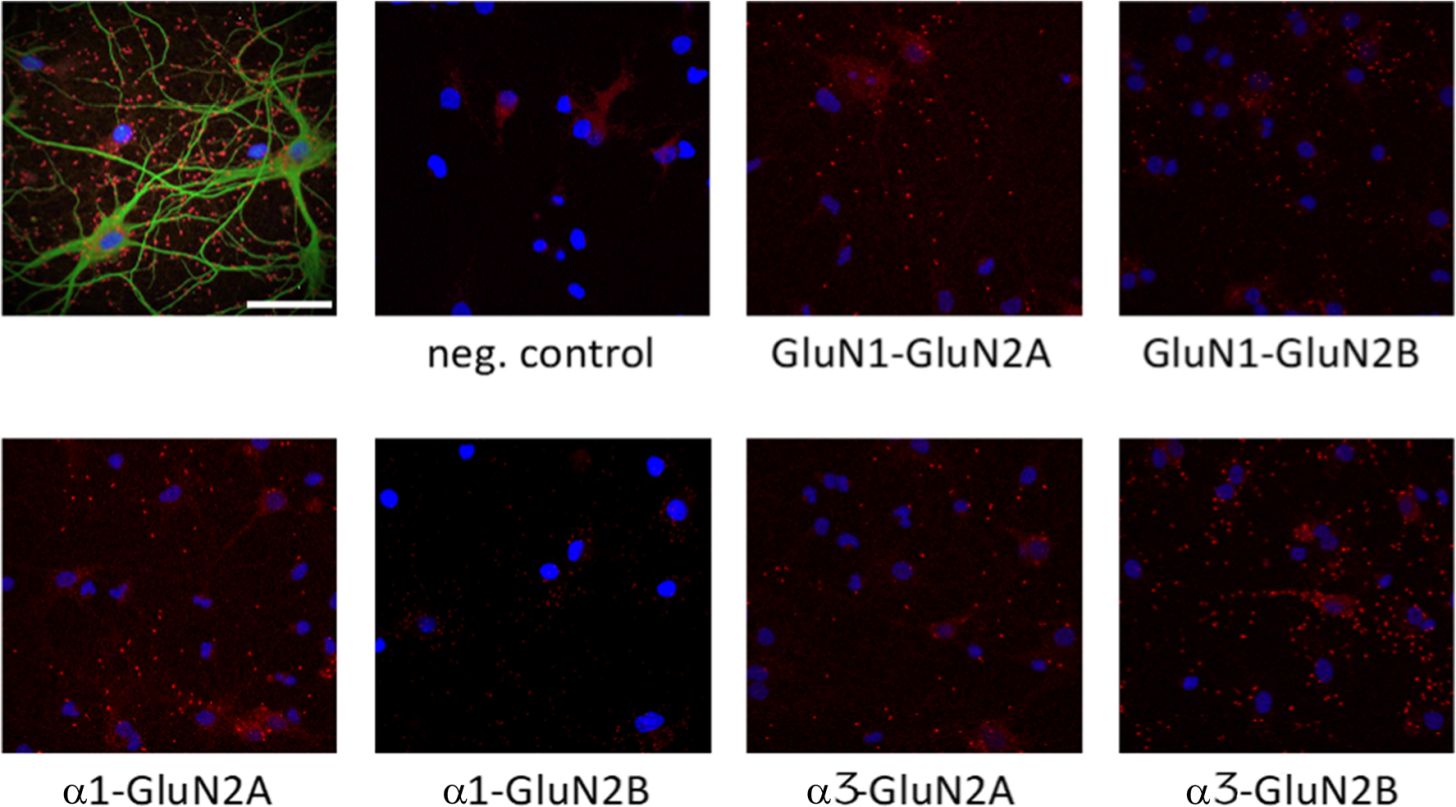
Proximity ligation assay (PLA) images of rat hippocampal neurons. PLA was performed using antibodies against Na,K-ATPase α-subunits (NKAα1 and NKAα3) and against GluN2-subunits (GluN2A and GluN2B). Omission of primary antibodies was used as negative control, and antibodies against GluN1 was used as a positive control. Red dots indicate PLA signal; cell nuclei are identified using DAPI stain. Upper left image is PLA of NKAα3 with GluN2B counterstained with pan neuronal marker in green to visualize neurons with extensions. Scale bar = 50 μm

To assess the possibility that NKA and NMDA receptor colocalize in hippocampal neurons, we first used proximity ligation assay (PLA), a robust and sensitive method that gives a signal for labeled proteins when they are within approximately 40 nm from each other [26–28]. By using antibodies against the NMDA receptor subunits GluN2A or GluN2B combined with the NKA ubiquitous α1 subunit or NKA neuron-specific α3 subunit, we detected a high density of PLA positive signal for all combinations of NMDA receptor and NKA antibodies (Fig. 5), indicating that NMDA receptor and NKA can form a complex in neuronal membranes.

**Fig. 6:**
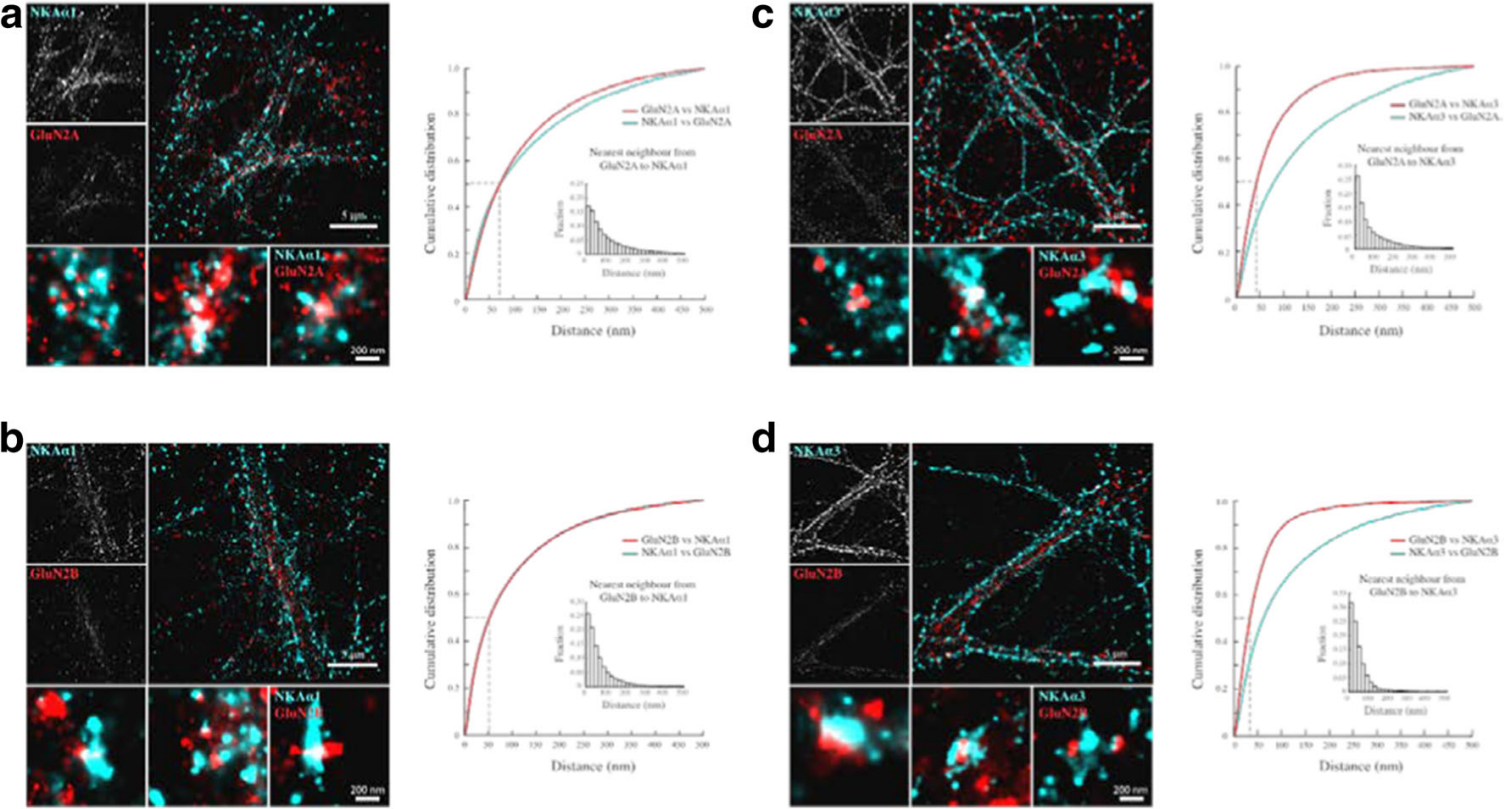
Super-resolution imaging shows that NMDAR and Na+,K + -ATPase are in close proximity in hippocampal neurons. **a**–**d** Overview images of dendritic segments from dSTORM experiments with antibody labeled GluN2-subunits (GluN2A and GluN2B) and Na,K-ATPase α-subunits (NKAα1 and NKAα3). The GluN2 antibodies were tagged with Alexa-647 conjugated secondary antibodies, and Na,K-ATPase antibodies were tagged with Atto-488 conjugated secondary antibodies. Images show Gaussian representations of clusters containing single localized molecules of dendritic segments and GluN2 subunit containing clusters. Cumulative probability distributions of quantified distances from GluN2 subunits to the closest Na,K-ATPase α and vice versa. Histogram insets show the relative frequency of distances from GluN2 subunits to the closest Na,K-ATPase α (insets). Scale bar = 5 μm (overviews of dendrites) and 200 nm (zoom in on clusters)

To further explore colocalization between NMDA receptor GluN2A and GluN2B subunits and the NKA α1 and α3 subunits on single dendrites, we used direct Stochastic Optical Reconstruction Microscopy (dSTORM). The NMDA receptor GluN2 subunits appeared to be distributed in relatively large clusters (Fig. 6). NKA α1 and α3 subunits displayed broader distributions along the postsynaptic membrane than the NMDA receptor GluN2 subunits but did also appeared as clusters. The distance between single molecules was analyzed using the nearest neighbor algorithm [29]. The analysis was performed on dendritic segments that were sparse with surrounding neurites, and the distances between all identified NMDA receptor GluN2A and GluN2B subunits and their nearest NKDA α1 and α3 subunits and between all identified NKA α1 and α3 subunits and their nearest NMDA receptor GluN2A and GluN2B subunit were determined. The cumulative distribution plots represent distances between each localized molecule and its closest neighbor within a 500-nm radius, which roughly corresponds to the size of a dendritic spine head. Both NKA α1 (Fig. 6a, b) and α3 (Fig. 6c, d) subunits were in close proximity to both the NMDA receptor GluN2A and GluN2B subunits. For 50% of the NMDA receptor GluN2A subunits, the distance to the nearest NKA α3 subunit was less than 50 nm, and to the nearest NKA α1 subunit, less than 70 nm. Since the measurement of the distance between the molecules also includes the size of the primary and secondary antibodies, our results are compatible with the conclusion that NMDA receptors are in close proximity with NKA and thus positioned to permit a direct interaction and modulation of the receptor function.

### Ouabain Attenuates NMDAR-Dependent Long-term Potentiation

**Fig. 7:**
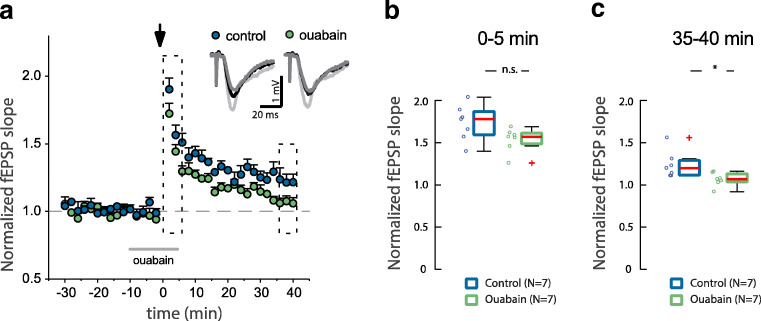
Ouabain decrease long-term potentiation in hippocampal CA3-CA1 synapses. **a** Time-course of changes in field EPSP (fEPSP) slopes after induction of LTP by theta-burst stimulation (arrow) in control (clear circles) and in 50 nM ouabain-treated rat hippocampal slices (filled circles). The solid gray bar denotes the time that ouabain was present in the perfused aCSF. Representative traces averaged fEPSP at baseline (dark gray) and after theta-burst stimulation (0–5 min, light gray; 55–60 min, black). **b**, **c** Bars summarizing the average changes (5 min) in fEPSP slopes after Ø-burst stimulation at the times indicated by dotted boxes in the time-course plot. n.s.—*p* > 0.05, *p < 0.05, Wilcoxon rank sum test

To investigate if the ouabain induced reduction in NMDA receptor calcium signal had an effect on synaptic function, we assessed the NMDA receptor–dependent long-term potentiation in hippocampal slices. Ouabain (50 nM) was added to the perfused aCSF for 10 min before the delivery of a theta-burst (Ø-burst) stimulation and was present during the first 5 min post-potentiation (Fig. 7a); this treatment leads to a significant decrease in the potentiated fEPSP slopes calculated at 35–40 min when compared with vehicle-treated slices. The concentration of ouabain was increased to 50 nM to assure that the drug reached the recording site at a high enough concentration. Treatment with ouabain did not affect the fEPSP baseline or the initial potentiation of fEPSP slopes induced by the Ø-burst stimulation protocol (Fig. 7b, c). Thus, a reduction in calcium influx mediated by ouabain affects the long-term synaptic plasticity.

## Discussion

Our study demonstrates that NKA and the NMDA receptor form protein complexes in hippocampal neurons and that application of nanomolar concentrations of the NKA-specific ligand ouabain to single neurons causes an immediate and rapidly reversible reduction of the calcium response to NMDA receptor activation. We have excluded that ouabain inhibition of the NMDA signal is caused by alteration of Src-dependent phosphorylation of the NMDA receptor or by internalization of NMDA receptor subunits. Our findings are compatible with the notion that ouabain-bound NKA can modulate NMDA receptor activity via protein-protein interactions.

Several previous studies have suggested a functional relationship between the NMDA receptor and NKA. Incubation of primary neurons with nanomolar ouabain concentration has been reported to decrease NMDA receptor expression within 1 h and incubation with NMDA for 30 min to decrease of Na,K-ATPase enzymatic activity [6]. Micromolar concentrations of ouabain has been reported to dose-dependently activate NFkB via an effect on NMDA receptor [30]*.* Song, Thompson, and Blaustein have reported that glutamate-evoked calcium signals can be augmented by pretreatment with ouabain in nanomolar concentration. This effect was blocked by inhibition of mGluR5 and the sodium/calcium exchanger, but not by the NMDA receptor inhibitor D-AP5 [31]. In our study, we used NMDA instead of glutamate to selectively stimulate the NMDA receptor. Sibarov et al. have reported that nanomolar concentrations of ouabain protects from the excitotoxic stress that accompanies prolonged activation of NMDA receptors [32]. In their study, the simultaneous application of NMDA 30 μM and ouabain 1 nM resulted in a calcium increase that gradually declined to almost control values. The ouabain effect was obvious approximately 5 min after application. In contrast, we recorded an immediate effect of ouabain on the NMDA response. The approximately ten times higher concentration of NMDA that was used in their study compared with what we used for perfusion might have contributed to the difference in results. Rodríguez de Lores Arnaiz and collaborators identified a cardiotonic steroid, endobain E, and reported that it was found to modulate the activity and expression of the NMDA receptor: It was suggested that endobain E interacted directly with the NMDA receptor [33–36]. However, since their studies were performed on crude synaptosomal membranes to exclude membrane depolarization and neurotransmitter release, it is likely that the membrane contained both NMDA receptors and NKA and that the effect can have been mediated via NKA bound endobain E.

Ouabain is known to activate Src kinase, which can phosphorylate other proteins including NMDA receptor. Inhibition of Src by PP2 did not abolish the ouabain-dependent modulation effect on NMDA-evoked calcium response. Ouabain is also known to activate a calcium signaling pathway characterized by slow onset and low-frequency calcium oscillations [14]. However, we found that the effect of ouabain on the NMDA receptor is instantaneous and rapidly reversible. Furthermore, our PLA and super-resolution microscopy studies showed that NKA and NMDA receptors are found in close proximity to each other in the neuron. Thus, we propose that ouabain exerts its effect directly on a NMDA receptor/NKA complex via a protein-protein interaction.

**Fig. 8:**
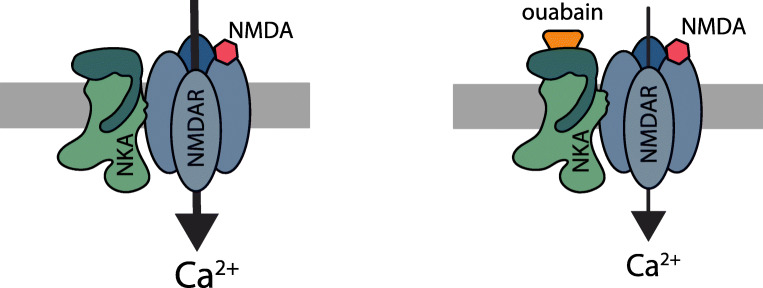
Ouabain induces a change in NKA/NMDA receptor protein-protein interaction and downregulates NMDA receptor–dependent calcium influx

Allosteric receptor-receptor interaction, which is still an underexplored mechanism of receptor modulation [37], has been suggested to be an especially effective approach to control NMDA receptor function [38–40]. The functional read-out from the interaction between two proteins is often modified by small molecules [41, 42], and it is well established that ouabain binding to the potassium-binding state of the catalytic NKA subunit changes its conformation [43–45]. Lack of effect of the low ouabain concentration on membrane potential (Supp. Fig. 3) and synaptic activity (Fig. 7) confirms that the ouabain effect is due to a modulation of the NMDA receptor, rather than to changes in NKA activity. Based on this background and our own results, we propose a model where a conformational change in the catalytic NKA subunits transfers to its interacting partner, the NMDA receptor, and reduces NMDA receptor activity (see scheme in Fig. 8).

The calcium permeability of the NMDA receptor confers a special role to this receptor through the calcium induced intracellular signaling. The NMDA receptor plays a crucial role in many brain functions, including learning and memory, but excess activation can cause excitotoxicity. The interest of NMDA receptor antagonists or modulators has increased recently, with the discovery that the NMDA receptor antagonist ketamine has an antidepressant effect at a low dose, and other NMDA receptor antagonists and modulators are currently being considered for antidepressant treatments [46]. Several cardiotonic steroids, including ouabain, have been found in mammalian organisms [9, 10]. Ouabain is produced in the hypothalamus [11]. There is as yet no evidence for a local neuronal release of ouabain or other cardiotonic steroids. The ouabain concentration in human cerebrospinal fluid has been reported to be 2 nM [47], indicating that the effects we recorded following application of ouabain 1–10 nM are physiologically relevant. The importance of the reduced NMDA function is further underscored by the fact that ouabain reduces late-LTP, consistent with the reduction in NMDA calcium currents that are instrumental in the establishment of late-LTP [48]. Taken together, the results from this study have revealed a potential role for endogenous cardiotonic steroids as physiological regulators of NMDA receptor function and opened up for further exploration of the pharmacological use of cardiotonic steroids to downregulate NMDA receptor activity.

## Material and Methods

### Cell Culture and Transfection

All animal experiments were approved by the Institutional Animal Care and Use Committee of the Karolinska Institutet. Primary hippocampal cultures were prepared from E18.5 Sprague Dawley rat embryos. Hippocampi were dissected, rinsed in HBSS with 20 mM HEPES, trypsinized (0.25% for 10 min at 37 °C), and mechanically dissociated in Minimum Essential Medium (Thermo Fisher Scientific) using a glass Pasteur pipette. Cells were seeded on 80 μg/ml poly-DL-ornithine (Sigma-Aldrich) coated #1.5 18 mm coverslips (Marienfeld) at a density of 10^4^/cm2.

Cells were allowed to attach on to coverslips in MEM with 10% horse serum (Thermo Fisher Scientific), 2 mM L-glutamine (Thermo Fisher Scientific), and 1 mM sodium pyruvate (Sigma-Aldrich). After 3 h, the media was changed to Neurobasal (Thermo Fisher Scientific) with 2% B-27 (Thermo Fisher Scientific), 2 mM L-glutamine, and 1% penicillin/streptomycin (Sigma-Aldrich). Experiments were performed on 21–24-day-old cultures.

Cultures were transfected 24–48 h prior to calcium imaging and internalization experiments using Lipofectamine 2000 (Thermo Fisher Scientific) according to the manufacturer’s protocol.

### Plasmids and Antibodies

Plasmids used in this study were pGP-CMV-GCaMP6f, a gift from Douglas Kim (Addgene plasmid #40755), and pCl-SEP_GluN2B, a gift from Robert Malinow (Addgene plasmid #23998).

Primary antibodies used in this study were mouse anti-Na,K-ATPase α1 (5.7 μg/ml, DHSB), mouse anti-Na,K-ATPase α3 (1 μg/ml, Thermo Fisher Scientific), mouse anti-GluN1 (1:1000, Millipore), rabbit anti-GluN2A (2 μg/ml for immunocytochemistry, Millipore), rabbit anti-GluN2B (2 μg/ml, Millipore), mouse anti-GluN2B (2.5 μg/ml for immunocytochemistry), rabbit anti-GluN2B-pY1252/1336/1472 (1:100, Phosphosolutions), rabbit anti-GFP (4 μg/ml, Thermo Fischer Scientific), and mouse anti-Pan neuronal marker (1:1000, Millipore).

Secondary antibodies used were goat anti-mouse-Atto 488 (5 μg/ml, Sigma-Aldrich Scientific), goat anti-rabbit-Alexa 647 (10 μg/ml, Thermo Fisher Scientific), goat anti-mouse-Alexa Fluor 488 (4 μg/ml, Thermo Fisher Scientific), donkey anti-rabbit-Alexa Fluor 568 (4 μg/ml, Thermo Fisher Scientific), goat anti-rabbit-Alexa Fluor 488 (4 μg/ml, Thermo Fisher Scientific), goat anti-rabbit-Alexa Fluor 568 (4 μg/ml, Thermo Fisher Scientific), and goat anti-mouse-STAR635P (1:200, Abberior). For PLA, anti-rabbit-PLUS (Sigma Aldrich) and Anti-Mouse-MINUS (Sigma Aldrich) were used according to manufacturer’s recommendations.

### Calcium Imaging

For the perfusion experiments calcium imaging was performed with Zeiss Axiovert 200 fluorescent microscope (Carl Zeiss), equipped with a 40x/1.3 NA oil immersion objective, and an C4742–95 CCD camera (ORCA-ERG, Hamamatsu) at 37 °C in a heated chamber. Excitation at 340 and 380 nm at 0.5 Hz during response and at 0.1 Hz during baseline recording was carried out with a monochromator (Polychrome IV, TILL Photonics). Devices were controlled, and data were recorded with the computer software MetaFluor (Molecular Devices).

Cells were loaded with 3.3 μМ fura-2-AM (Invitrogen) for 30 min at 37 °С in HEPES buffer (160 mM NaCl, 5.4 mM NaCl, 1.3 mM CaCl_2_*2H_2_O, 0.81 mM MgSO_4_*7H_2_O, 0.78 mM, NaH_2_PO_4_*2H_2_O, 20 mM HEPES, 20 mM D-glucose, pH 7.4). Cover glass with cells was mounted in a temperature-controlled chamber, and perfused with aCSF solution heated to 37 °C (125 mM NaCl, 26 mM NaHCO_3_, 4 mM KCl, 0,5 mM MgSO_4_*7H_2_O, 1,25 mM NaH_2_PO_4_*H_2_O, 2 mM CaCl_2_*2H_2_O), constantly bubbled with 5% CO_2_, 95% O_2_ to maintain pH 7.4. After sampling baseline fluorescence for approximately 5 min, cells were perfused with different ligand solutions, 4 μM NMDA, for 2 min, and after restoration of baseline Ca^2+^ levels treated again with either 4 μM NMDA or 4 μM NMDA with different ouabain concentrations. In the end of each experiment, 100 μM NMDA was perfused to distinguish between neuronal and glial cells. An experiment was run for a maximum of 35 min. The intensity of somatic fura-2 fluorescence was extracted from MetaFluor software and further analyzed in Matlab. The response to treatment was quantified as the normalized peak amplitude of fura-2 signal. The peak amplitude was defined as the difference between baseline, established during 5 min before treatment, and the maximal signal during treatment and finally normalized to the peak response at first treatment (4 μM NMDA).

For local stimulation experiments, calcium imaging was performed on a Zeiss Observer.D1 inverted widefield microscope (Carl Zeiss) equipped with a 40x/1.3 NA oil immersion objective, an iXon +897 EMCCD camera (Andor Technology), an X-cite excite lamp (Lumen Dynamics), and a GFP excitation/emission filter set. Imaging was done at 1 Hz with an exposure time of 5 ms. During experiments, GCaMP6f-transfected cells were kept in Mg2+ free Krebs Ringer Buffer (Krebs), consisting of 111 mM NaCl, 4 mM KCl, 1 mM NaH_2_PO_4_·H_2_O, 25 mM NaHCO_3_, 1.5 mM CaCl_2_·H_2_O, 10 mM D-glucose, and 20 mM HEPES, pH 7.4 at ~37 °C. After sampling baseline GCaMP6 fluorescence for 2 min, single neurons were treated locally for 20 s using a multi-well microfluidic pipette (Biopen, Fluicell). The pipette was centered adjacent to the soma to stimulate the soma and proximal dendrite. In studies presented on Figs. 2a–c and 3b, c, a paired protocol was used, and cells were treated with solutions loaded into different wells of the same pipette. In the other studies presented on Fig. 2d, the experiments were unpaired and individual pipettes were used for different treatments. Average intensities of somatic GCaMP6f fluorescence in treated cells were extracted in Zen 2010 software and further analyzed in Matlab. Averages of the normalized intensity, ΔF/F0, in the soma were extracted and further analyzed in Matlab, where ΔF/F0 represents changing of fluorescence (ΔF) normalized to baseline F0.

### Super-Resolution Localization Microscopy

Primary neurons were first fixed in 4% paraformaldehyde (Sigma-Aldrich) for 10 min at room temperature and then with 10% trichloroacetic acid (Calbiochem) for 10 min at 4 °C, rinsed and permeabilized in 0.1% Triton X-100 (Sigma-Aldrich) for 2 min. Blocking was done using 10% normal goat serum (NGS, Jackson Laboratory).

Cells were incubated with primary antibodies pairwise using mouse anti-Na+, K+-ATPase α1 or mouse anti-Na+, K+-ATPase α3 and rabbit anti-GluN2A or rabbit anti-GluN2B in 5% NGS for 1 h at room temperature or overnight at 4 °C. After repeated rinsing, cells were incubated with goat anti-mouse-Atto 488 and goat anti-rabbit-Alexa 647 for 1 h and then rinsed repeatedly.

During imaging sessions, cells were kept in a buffer consisting of 50 mM Tris-Cl, pH 8.0, 10 mM NaCl and 10% (w/v) glucose with an oxygen scavenging system made of 0.4 mg/ml glucose oxidase (Sigma-Aldrich), 70 μl catalase from bovine liver (Sigma-Aldrich), and 10 mM cysteamine (Sigma-Aldrich). The stock solutions of glucose oxygen + catalase and cysteamine were kept at 4 °C and used for a maximum of 2 weeks after preparation. The final imaging buffer was prepared fresh and replaced within 2 h.

Single molecule imaging was performed on a Zeiss Elyra (Carl Zeiss) equipped with a Plan-Apochromat 100x/1.46 Oil objective and an Andor iXon EM-CCD. A single laser line of 488 nm (200 mW, back focal plane of the objective 50 mW, set at 100%) or 642 nm (150 mW, back focal plane of the objective 25 mW, set at 100%) is adequate to activate, excite, and deactivate Atto-488 and Alexa-647, respectively. A low-wavelength 405-nm laser (50 mW, back focal plane of the objective 10 mW, set at 1%) was continuously used to further help push dye molecules into an activated state [49]. Detection was done using a 495–550 nm bandpass filter for Atto-488 and a 655 long pass filter for Alexa-647.

Series of 20,000–25,000 images of each of the fluorescent dyes in a dendritic segment were acquired with a pixel width of 100 nm and an 18 ms exposure time. The localization of individual fluorescent molecules was done in ZEN 2010 (Carl Zeiss) software with the following constraints: discard overlapping molecules, #photons > 200, 0.5 < chi square < 2, 10 nm < precision<50 nm.

The localizations were extracted and further analyzed in Matlab. After an autocorrelation-based approach to compensate for drift within and between channels, the distance to the closest localized Na,K-ATPase α-isoform to every GluN2 subunit, and vice versa, was determined by nearest neighbor analysis. Images to visualize distributions were rendered in in Zen 2010 with a pixel resolution of 10 nm and a Gaussian smoothing of 0.75× point spread function.

### Proximity Ligation Assay

Coverslips with primary neurons were first in cold 10% trichloroacetic acid (Calbiochem) for 10 min at 4 °C, rinsed in PBS and permeabilized in 0.1% Triton X-100 (Sigma-Aldrich) for 90 s. The Sigma DuoLink In Situ Red Kit Mouse/Rabbit was used according to manufacturer’s instructions.

Briefly, cells were blocked for 60–90 min and then incubated with primary mouse antibodies against Na,K-ATPase α (a6f), anti-Na,K-ATPase α3 (MAB), or mouse anti-GluN1 together with rabbit antibodies against either GluN2A or GluN2B. Antibodies were diluted in DuoLink Antibody Diluent and incubated with coverslips overnight at 4 °C. Duolink PLA-probes, constituting of an antibody and fused oligonucleotide probe, were diluted in Antibody Diluent solution. We used anti-mouse (MINUS) respective anti-rabbit-(PLUS) probes, which were incubated for 1 h at 37 °C.

After washing, the oligonucleotides fused to the secondary antibodies were ligated together by DuoLink Ligase in DuoLink Ligase Buffer (1×) for 30 min at 37 °C. If the secondary antibodies are within 40 nm from each other, they can be successfully ligated. Next, the signal from ligated oligonucleotides was amplified for 100–120 min at 37 °C, using DNA polymerase in polymerization buffer. The coverslips were washed in buffer B and mounted in Duolink In Situ Mounting Medium with DAPI and stored at − 20 °C. Images were recorded with Zeiss LSM 510 Meta scanning confocal microscope using 63x/1.40 Oil DIC. PLA signal was detected using 543 nm excitation and a LP 560 filter and DAPI using 405-nm excitation and a 420–480-nm band-pass filter.

### Confocal Microscopy

A Zeiss 780 confocal laser scanning microscope (Carl Zeiss) equipped with a Plan-Apochromat 63X/1.4 oil objective was used to analyze samples from the internalization and phosphorylation experiments. Excitation was done with 488 nm (for Alexa 488), 561 nm (for Alexa 568), and 633 nm (for STAR635P) laser lines. Emission was detected at bandwidths of 493–574 nm (for Alexa 488), 579–633 nm for (Alexa 568) (574–712 for dual color imaging), and 651–758 nm (for STAR635P). In the phosphorylation experiments, the pinhole was set to 2.5 μm and the pixel width to 66 nm. In the internalization experiments, the pinhole was maximally open and the pixel width 89 nm. Within experimental groups microscope settings were identical.

### Electrophysiology

Whole-cell patch clamp recordings were performed on primary hippocampal cells at DIV 16–21. Cultures were maintained in 110 mM NaCl, 4 mM KCl, 1 mM NaH_2_PO_4_, 25 mM NaHCO_3_, 1.5 mM CaCl_2_, 1.2 mM MgCl_2_, 10 mM glucose, and 20 mM HEPES at pH 7.4 at 37 °C. The internal pipette solution contained 120 mM K^+^-gluconate, 24 mM KCl, 4 mM NaCl, 4 mM MgCl_2_, 0.16 mM EGTA, 10 mM HEPES, 4 mM K_2_-ATP, pH 7.2 adjusted with KOH. After entering whole-cell configuration, cells were perfused with Mg^2+^-free Krebs with and without ouabain, ouabain concentrations ranging from 1 nM, 10 nM, 100 nM, to 1 μM, respectively. The experiments were done at 32 °C, the flow rate of the perfusion system was 2 mL/min, and each solution had a perfusion duration of 4 min. Recordings were performed using an Axopatch 200B amplifier (Molecular Devices) and pClamp software (Version 8.2, Molecular Devices).

### GluN2B Phosphorylation

An immunofluorescence-based assay was used to study specific phosphorylation of the three tyrosine phosphorylation sites of GluN2B: pY1252, pY1336, and pY1472. Primary hippocampal cells were treated with Krebs, 10 nM ouabain, dimethyl sulfoxide (DMSO, Sigma-Aldrich, 1:5000), or 10 μM PP2 (Sigma-Aldrich) in DMSO (1:5000) for 5 min at 37 °C. After treatment, cells were immediately fixated in 4% paraformaldehyde for 10 min. Cells were rinsed, permeabilized in 0.1% Triton X-100 (Sigma-Aldrich) for 2 min, and subsequently incubated in 10% normal goat serum (NGS, Jackson Laboratory) blocking solution for 1 h. Primary antibody incubation was done at room temperature for 1 h or at + 4 °C overnight with a mouse antibody for total GluN2B in pair with either the rabbit phosphor-specific antibodies against the GluN2B subunit Tyr1252, Tyr1336, or Tyr1472 in 5% NGS. After repeated rinsing, cells were incubated with goat anti-mouse-Alexa Fluor 488 and donkey anti-rabbit-Alexa Fluor 568 antibodies in 5% NGS for 1 h at room temperature. Cells were rinsed thoroughly, and the coverslips mounted in Prolong gold (Thermo Fisher Scientific). The mounting media was allowed to polymerize for at least 48 h before imaging sessions.

### NMDA Receptor Internalization

Primary neurons transfected with recombinant GluN2B tagged to superecliptic pHluorin (GluN2B-SEP) were live labeled with a rabbit GFP tag antibody (1:500, Thermo Fisher Scientific) in Neurobasal media (Thermo Fisher Scientific) for 30 min at + 4 °C. Cells were then treated with either Krebs (+Mg^2+^), 10 μM NMDA, or 10 μM NMDA+10 nM ouabain in Mg^2+^ free Krebs for 5 or 15 min at + 37 °C and immediately fixated in 4% paraformaldehyde for 10 min. Nonspecific antibody binding to surface proteins was reduced by incubating cells with 10% NGS for 30 min. As pHluorin was heavily quenched after fixation and GFP antibody binding, the surface pool of GFP-labeled GluN2B-SEP was labeled with goat anti-rabbit-Alexa Fluor 488 for 1 h in 5% NGS. Available GFP-epitopes on the cell surface was blocked using excessive amounts of goat anti-rabbit AffiniPure F(ab’)_2_ fragment (0.13 mg/ml, Thermo Fisher Scientific) for 2 h.

Cells were then permeabilized with 0.1% Triton X-100 for 2 min. After an additional blocking step with 10% NGS, the pool of GluN2B that was internalized after treatment was labeled with goat anti-rabbit-Alexa Fluor 568 for 1 h. Simultaneously, cells were incubated with a neuronal marker (mouse Pan Neuronal antibody) for identification and morphology. After repeated rinsing, cells were incubated with goat anti-mouse-STAR635. Cells were rinsed repeatedly, and the coverslips were mounted in Prolong Gold and left to dry for at least 48 h before imaging.

A machine learning algorithm-based software (Ilastik) was trained on a subset of images to automatically identify surface tagged GluN2B using the Alexa 488 channel. After a training session on 5 images, the complete data set was processed and the output data set to binary images that could further be used as masks. The average intensity of internalized GluN2B in the Alexa 568 channel was then extracted in Matlab, by using the Alexa 488 binary mask images. Finally, the endocytotic index was calculated as the mean intensity of Alexa 568 normalized to Alexa 488 mean intensity.

### Electrophysiological Studies of Brain Slices

Experiments were performed in accordance with the ethical permit granted by Norra Stockholms Djurförsöksetiska Nämnd (N13/15). Sprague Dawley male rats (4- and 5-week-old) from Charles River Laboratories were used in all the experiments. Rats were deeply anesthetized using isoflurane and decapitated soon after the disappearance of corneal reflexes. Brain was removed and placed in ice-cold standard artificial CSF (aCSF) containing in mM: 124 NaCl, 30 NaHCO_3_, 10 glucose, 1.25 NaH_2_PO_4_, 3.5 KCl, 1 MgCl_2_, and 2 CaCl_2_. Hippocampal horizontal sections (400 μm thick) were prepared using a Leica VT1200S vibratome (Leica Microsystems). Immediately after slicing, sections were transferred into an interphase incubation chamber filled with standard aCSF. The chamber was held at 34 °C during the slicing period, and it was subsequently allowed to cool-down at room temperature. After a recovery period of 2 h, slices were transferred to a submerged recording chamber with perfusion rate 2–3 ml per min with standard aCSF at 32 °C, bubbled with carbogen gas (5% CO_2_, 95% O_2_).

Long-term potentiation (LTP) was monitored in the Shaffer collaterals (SC)-CA1 pathway, for this purpose, an extracellular recording pipette filled with regular aCSF was placed in the stratum radiatum and field excitatory postsynaptic potentials (fEPSPs) were evoked by electrical stimulation of SC using a bipolar concentric electrode. Stable fEPSP baseline responses were collected every 30 s during at least 30 min using 50–60% of the maximal response. To induce LTP in the CA1 region, a theta-burst stimulation (Ø-burst) protocol was delivered through the stimulation electrode, a Ø-burst consisted in 2 trains with 10 bursts of 4 pulses at 100 Hz; bursts were delivered at 5 Hz. Ouabain-containing solutions were protected from light, and experiments were carried out in a dark room. An ouabain stock was freshly prepared at 1 mM concentration and dissolved in water (36 °C for 90 min).

### Statistical Analysis

Statistical significance was determined by Wilcoxon rank sum test for independent samples and Wilcoxon signed-rank test for paired samples. For multiple groups, one-way ANOVA with post hoc Bonferroni test or repeated measures ANOVA were used.

## Electronic Supplementary Materials

ESM 1 (AVI 47439 kb)

ESM 2 (DOCX 1546 kb)
