# Supplementary material for: Ouabain Modulates the Functional Interaction Between Na,K-ATPase and NMDA Receptor
Source: Mol Neurobiol. 2020 Jul 10;57(10):4018–30. doi: 10.1007/s12035-020-01984-5 (PMC7467916; doi:10.1007/s12035-020-01984-5)
Supplement: Supplementary file 2 — (DOCX 1546 kb) [file 12035_2020_1984_MOESM2_ESM.docx]

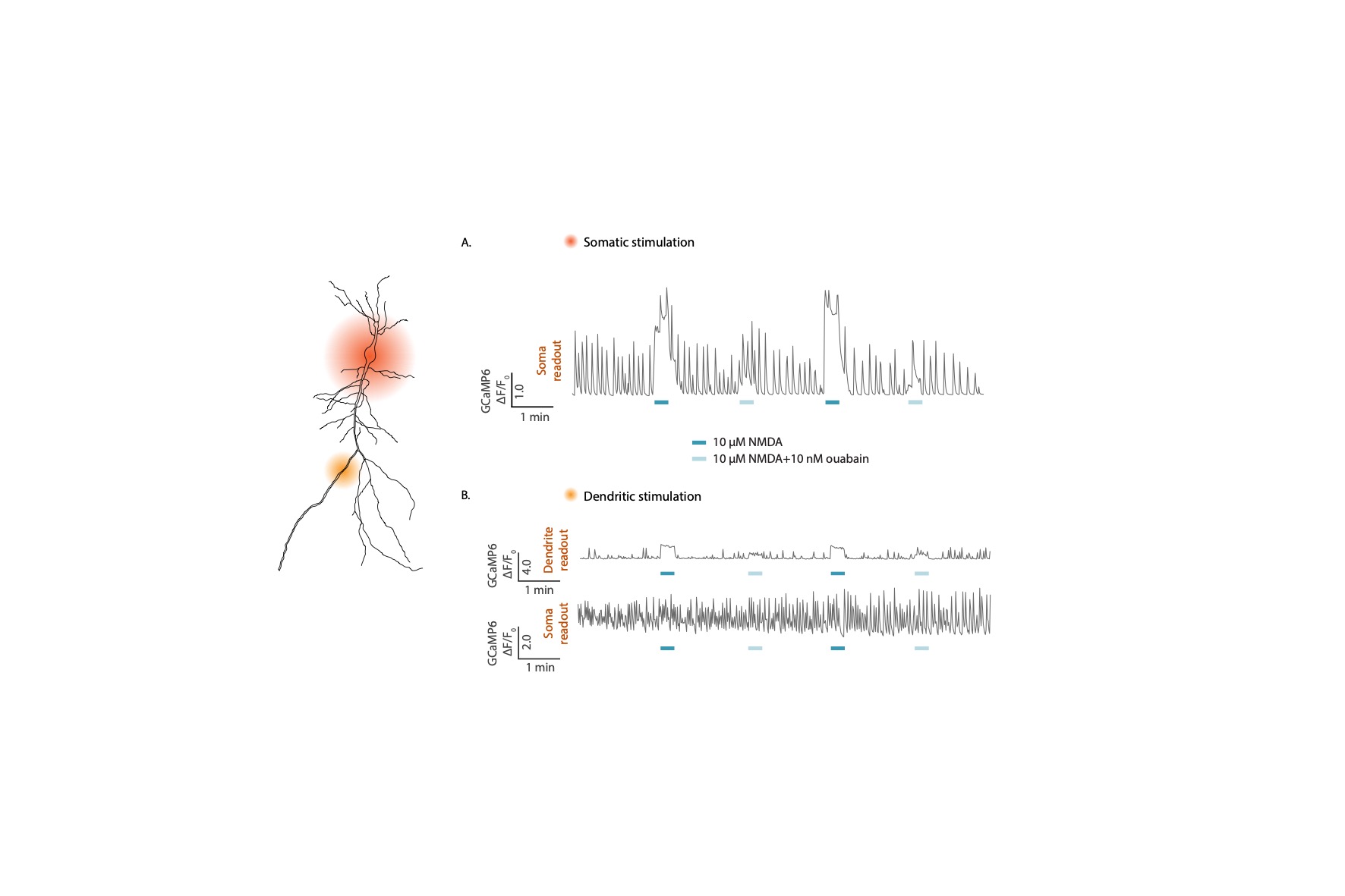


**Supplementary Figure 1. The effect of ouabain is reversible and locally produced**. A. The effect of ouabain is reversible. The calcium response in a neuron exposed to 10 μM NMDA, 10 μM NMDA+10 nM ouabain, 10 μM NMDA and 10 μM NMDA+10 nM ouabain during 20 s. B. The effect is locally produced. The calcium trace from a dendrite (top) and soma (bottom) in response to local application of NMDA and ouabain as in A on the dendrite during 20 s.

**Supplementary Figure 2. Ouabain alone did not cause changes in resting calcium level.** Traces of calcium responses in cells exposed to NMDA (10 μM), NMDA+ouabain (10 nM), ouabain (10 nM) and ouabain (1 μM) for 20s.

**Supplementary Figure 3. Membrane potential is unaffected by short-term application of nanomolar concentrations of ouabain**. A, A typical trace of the membrane potential from a whole-cell patch clamp recording in a primary cultured hippocampal neuron perfused with increasing concentrations of ouabain: vehicle, 1 nM, 10 nM, 100 nM, 1 μM and 50 μM. Cells were perfused with each concentration of ouabain for 4 min. B, Boxplots showing the resting membrane potential at increasing concentrations of ouabain (* - p<0.05, ** - p<0.01, repeated measures ANOVA). N = 9.

**
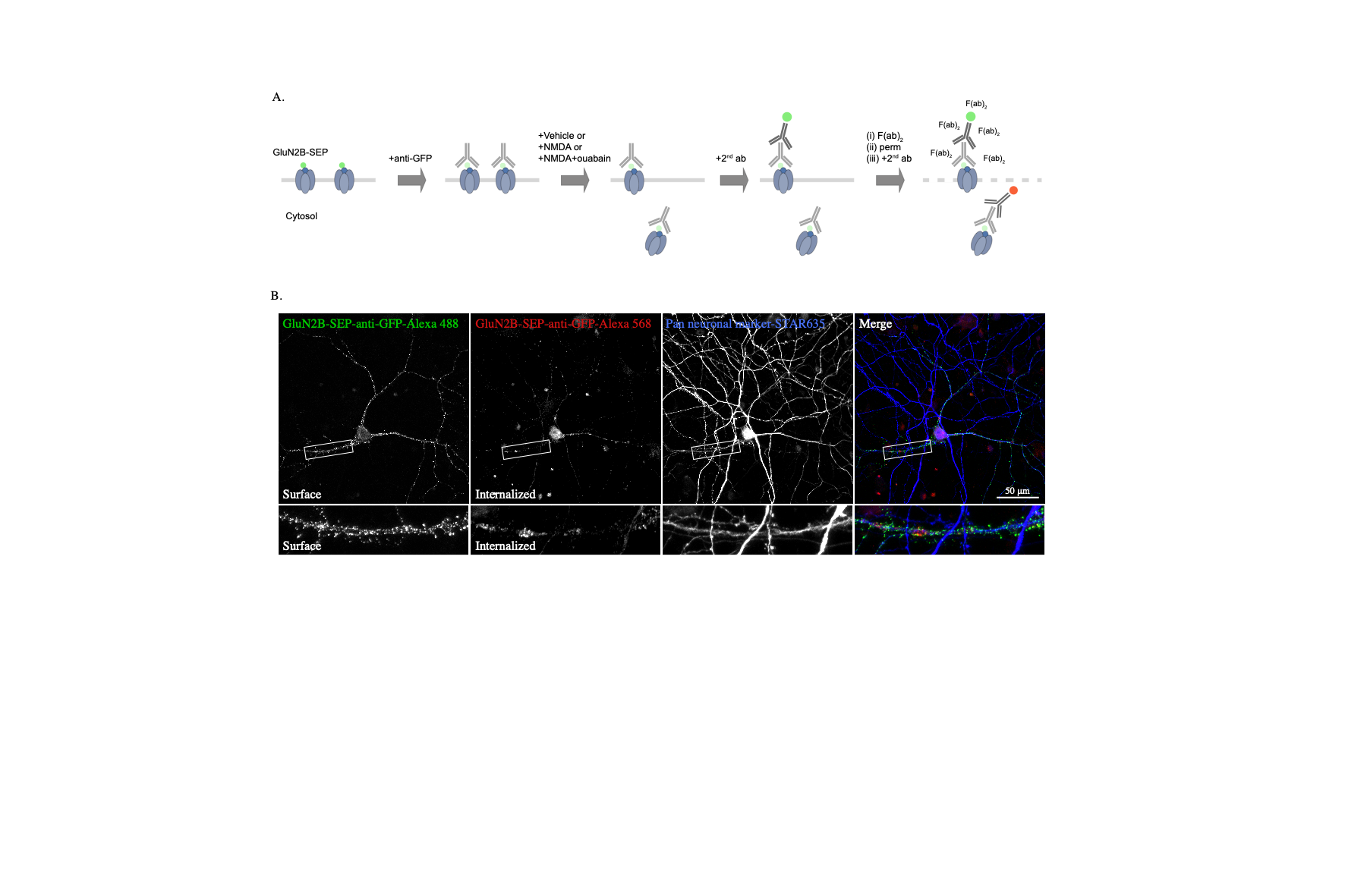
**

**Supplementary Figure 4. Internalization protocol description**. A, An illustration of the antibody feeding assay employed to evaluate rapid GluN2B internalization. B, Representative images from antibody feeding experiments in neurons transfected with GluN2B-pHluorin. Anti-GFP-Alexa-488 showing surface localized GluN2B subunits, anti-GFP-Alexa-568 showing internalized GluN2B subunits and Pan neuronal marker-STAR635P revealing the neuronal morphology. Scale bar = 50 μm.
